# Ocular herpes: the pathophysiology, management and treatment of herpetic eye diseases

**DOI:** 10.1007/s12250-014-3539-2

**Published:** 2014-12-15

**Authors:** Lucy Zhu, Hua Zhu

**Affiliations:** Department of Microbiology, Biochemistry & Molecular Genetics Rutgers, Rutgers — New Jersey Medical School, Newark, New Jersey 07103 USA

**Keywords:** ocular herpes, herpetic eye diseases, varicella zoster virus (VZV), cytomegalovirus (CMV), herpes simplex virus (HSV)

## Abstract

Herpesviruses are a prominent cause of human viral disease, second only to the cold and influenza viruses. Most herpesvirus infections are mild or asymptomatic. However, when the virus invades the eye, a number of pathologies can develop and its associated sequelae have become a considerable source of ocular morbidity. The most common culprits of herpetic eye disease are the herpes simplex virus (HSV), varicella zoster virus (VZV), and cytomegalovirus (CMV). While primary infection can produce ocular disease, the most destructive manifestations tend to arise from recurrent infection. These recurrent infections can wreck devastating effects and lead to irreversible vision loss accompanied by a decreased quality of life, increased healthcare usage, and significant cost burden. Unfortunately, no method currently exists to eradicate herpesviruses from the body after infection. Treatment and management of herpes-related eye conditions continue to revolve around antiviral drugs, although corticosteroids, interferons, and other newer therapies may also be appropriate depending on the disease presentation. Ultimately, the advent of effective vaccines will be crucial to preventing herpesvirus diseases altogether and cutting the incidence of ocular complications.

## INTRODUCTION

Certain sites in the human body are immune-privileged, meaning that they have evolved to tolerate the introduction of antigens without provoking an inflammatory immune response. This phenomenon protects vital organs and their functions from the damaging effects of inflammation. The eye is one example of an immune-privileged site. Ocular tissues have minimal ability to regenerate if damaged, and as such, each structure within the eye has developed a variety of overlapping molecular and cellular mechanisms to suppress potentially destructive T-cell responses and maintain tissue integrity (Hori J, 2008; Stein-Streilein J, 2008). While these mechanisms limit local tissue destruction to help preserve vision, they also render the eye more vulnerable to infections that require a more robust inflammatory response for eradication (Dartt D A, 2011).

Ocular infections caused by herpesviruses present a serious clinical problem in both industrialized countries and developing nations around the world. While there are over 25 viruses in the *Herpesviridae* family, only eight are able to produce infection in humans. The most common culprit of herpetic eye disease is the herpes simplex virus. While both herpes simplex virus-1 and -2 (HSV-1 and HSV-2) can elicit ocular disease, HSV-1 is far more likely to spread to the eyes, causing infection and inflammation of the cornea referred to as herpes simplex keratitis. HSV keratitis is the most frequent type of keratitis and is also one of the leading causes of blindness around the world (Karsten E, et al., 2012). Globally, the incidence of HSV keratitis is roughly 1.5 million, including more than 40,000 new cases of severe monocular visual impairment each year (Farooq A V, et al., 2012). In the United States alone, 500,000 people are estimated to have ocular HSV, with treatment of new and current cases costing the country $17.7 million annually (Lairson D R, et al., 2003; Liesegang T J, et al., 1989).

Another herpesvirus associated with ocular disease is the varicella zoster virus (VZV). VZV is the causative agent of varicella and zoster, or better known as chickenpox and shingles, respectively. In the eye, VZV can instigate an extensive list of ocular manifestations stemming from herpes zoster ophthalmicus (HZO), with the more serious sequelae including chronic ocular inflammation, vision loss, and debilitating pain. These complications often times result in a decreased quality of life and considerable health care use. A mean of 10.8 eye visits per HZO patient was recently reported with the average eye-related complication lasting over 300 days (Yawn B P, et al., 2013).

Cytomegalovirus (CMV) can cause eye disease in immunocompromised individuals. While CMV retinitis is the most common intraocular disease in patients with acquired immunodeficiency syndrome (AIDS), the disease is also becoming more prevalent in patients with normal immune function. If left untreated, CMV retinitis can lead to progressive destruction of retinal tissue and irreversible visual loss. In fact, CMV retinitis is the leading cause of blindness in AIDS patients and in many developing countries (Yen M, et al., 2014). Lastly, the Epstein-Barr virus (EBV), human herpesvirus-6 (HHV-6) and human herpesvirus-8 (HHV-8) can also elicit ocular disease on rare occasion. This review will go into more detail about the pathophysiology of herpetic eye diseases as well as current management strategies, with a focus on the main culprit of ocular herpes, the herpes simplex virus.

## HERPES SIMPLEX VIRUSES

Herpes simplex virus (HSV) is a double-stranded DNA virus belonging to the *Alphaherpesvirinae* subfamily of *herpesviruses*. HSV exists as two types, type 1 (HSV-1) and type 2 (HSV-2), also known as human herpesvirus 1 (HHV-1) and human herpesvirus 2 (HHV-2), respectively. These two viruses are highly related, sharing nearly 70% genomic homology, but can be differentiated by antigenic differences in their envelope proteins. Not surprisingly, HSV-1 and HSV-2 also have very similar characteristics. Both herpesviruses contain a large genome that encodes over 80 proteins. Many of these proteins function in host cell interaction and immune evasion, helping the two viruses bind, fuse and spread from cell to cell during infection (Murray P R, et al., 2005). Symptomatic disease caused by HSV-1 is typically limited to keratitis in the eyes and herpes labialis (cold sores) on the mouth. HSV-2, on the other hand, is almost entirely associated with genital disease, commonly genital herpes. However, both HSV-1 and HSV-2 have the ability to produce disease on identical regions of the body (Akhtar J, et al., 2009).

### Epidemiology

The infection rates of HSV-1 and HSV-2 vary largely across geographical regions and are influenced by factors such as location, race, age, sex, and social class. In a 2002 study, the HSV-1 seroprevalence in adult populations of the United States, Germany, and Tanzania was determined to be >50%, >75% and >90%, respectively, with the worldwide seroprevalence estimated to be between 80%-90% (Rabenau H F, et al., 2002; Smith J S, et al., 2002). Interestingly, a general decline in HSV-1 prevalence has been noted in many industrialized nations. This may be a result of improved hygiene and better living conditions, leading to reduced exposure to the virus (Bradley H, et al., 2014; Farooq A V, et al., 2012; Steiner I, et al., 2007).

While HSV-1 infections are dropping in the United States, conflicting data exists regarding the infection rates of HSV-2. Certain seroprevalence studies have found increases in HSV-2 rates rising to 22% in the US, while others have noted either an overall decline to 17% or no noticeable changes over the last decade (Bradley H, et al., 2014; Xu F, et al., 2006). In Europe, HSV-2 infection rates are reportedly between 4%-24% and in some developing countries, over 60%. Generally speaking, the worldwide seroprevalence of HSV-2 is around 20% with HSV-2 infections occurring more frequently in women (Azwa A, et al., 2009).

HSV is present in lesions and can also be found in body fluids such as saliva and vaginal secretions. The notion that HSV-1 and HSV-2 can be distinguished by their route of transmission is a widely thought misconception. Although HSV-1 is typically transmitted via the orolabial route (e.g. kissing) and HSV-2 is typically transmitted via sexual contact or maternal transmission to newborns during childbirth, this is not always the case. It is actually now clear that recurrent oral herpes can be caused by HSV-2 (Buxbaum S, et al., 2003). Recent studies have also confirmed that a significant portion of genital herpes in developed countries is attributable to HSV-1, likely due to an increase in oral-genital contact mixed with the decline in childhood HSV-1 transmission, hence lower immunity to genital HSV-1 infection (Nilsen A, et al., 2000; Scoular A, et al., 2002; Tran T, et al., 2004).

Even though both HSV-1 and HSV-2 are capable of infecting identical sites on the body, HSV-2 will very rarely cause ocular disease in healthy adults. Cases of HSV-2 ocular disease are generally found in neonates who have acquired the virus in utero, during delivery, or postnatally from an infected individual. Because of their underdeveloped immune system, the subsequent infection can be severe and even lead to death. Owing primarily to HSV-1 instead, there are about 50,000 new and recurring diagnosed cases of ocular HSV in the United States each year. On a global scale, an estimated 10 million individuals have had ocular HSV, of which approximately two million are left with impaired vision (Wilhelmus K R, 2010).

## Infection and reactivation

HSV infection begins with direct contact with an individual who is shedding the virus. Virions enter the new host via mucosal surfaces or damaged skin at the site of contact. The viral envelop fuses with the plasma membrane of the target cell during entry. This utilizes concerted action from many HSV glycoproteins and is initiated specifically by the interaction of glycoprotein-D with its receptor. Adding to the complexity of HSV infection, the virus also demonstrates alternate routes of entry, including manipulation of a phagocytosis-like pathway to enter via an intracellular vesicle (Akhtar J, et al., 2009; Toma H S, et al., 2008). Immediately following entry, HSV shuts off the host's cellular protein synthesis processes by degrading host mRNA molecules (Kwong A D, et al., 1987). In the two to four hours post infection, the virus undergoes an acute phase during which replication rapidly occurs. Lesions form as the virus multiplies in the host epithelial cells, inducing eventual lysis before spreading to adjacent cells. After a round of replication, the virus travels by retrograde axonal transport to the sensory nerve ganglia where it establishes latency in neuronal cells (Al-Dujaili L J, et al., 2011). During the latent infection, viral DNA and RNA transcripts are present, but no infective viral particles are produced. The virus still retains its potential to reactivate, resume replication and cause recurrent disease; a characteristic shared by all herpesviruses. Because of this, HSV-1 and HSV-2 infections are considered to be persistent infections, having the ability to continuously infect others for life.

Reactivation from this latent state is triggered by physiological, chemical and environmental stressors. During reactivation, the virus travels back down the nerve axon to the body's surface where it replicates and causes recurrent disease. HSV can recur at the original site of infection or at any other site that is innervated by the infected ganglion. Typically though, the virus will surface in areas dense with sensory receptors, such as the cornea, oral mucosa, lips and fingertips (Toma H S, et al., 2008). Viral shedding is responsible for the ulcerative lesions seen in individuals presenting with clinical symptoms, although asymptomatic shedding can also take place in those with a subclinical infection. In both situations, this viral shedding allows for the transmission of HSV to a new host. Replication lasts five to six days, after which the virus returns to its latent stage and lesions heal.

## Pathophysiology of ocular herpes

When the initial HSV infection causes disease in the eye, it is termed primary ocular herpes. Primary ocular infections arise predominantly from direct inoculation on the surface of the eye, but in immunocompromised individuals, can be the result of autoinoculation from an active HSV infection elsewhere on the body (Gimenez F, et al., 2013). Primary ocular infections can also occur through HSV infection in the orofacial region, with the virus traveling through the ophthalmic branch of the trigeminal ganglion into the eyes.

More commonly, involvement of the eye comes from the reactivation of latent HSV. The virus usually establishes latency following a non-ocular route of infection, such as acquisition from close contact with active orolabial lesions, virus-laden saliva or lesions on the genital mucosa. The predominant site of infection is the orofacial region innervated by the maxillary branch of the trigeminal nerve (Figure 1). In this scenario, the virus then travels up the maxillary branch and rests during latency. Upon reactivation, HSV will spread to the ophthalmic division of the trigeminal ganglion, where it enters the basal epithelium and emerges at the corneal surface causing viral shedding (Ohara P T, et al., 2000; Rowe A M, et al., 2013; Tasman W J E A, 2013). Reactivation tends to elicit the first clinically apparent episode of ocular herpes,

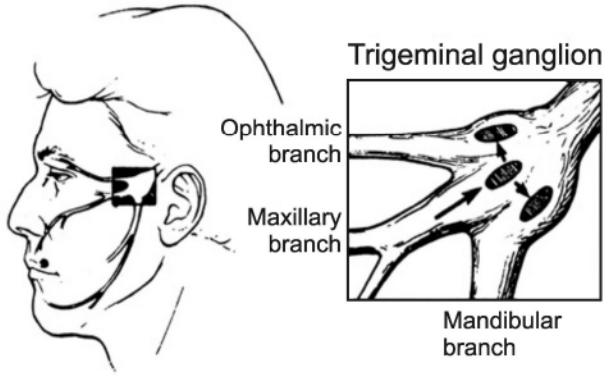

Figure 1. The trigeminal ganglion supplies neuronal innervation to most of the head and has three major branches: the ophthalmic branch, maxillary branch, and mandibular branch. Most commonly, infection of HSV-1 occurs in the orofacial region served by the maxillary branch. During primary infection or reactivation, the virus can spread to the ophthalmic branch, leading to infection of the ophthalmic division of the trigeminal ganglion and subsequent ocular disease (Liesegang, 1992) (Reuse with permission).

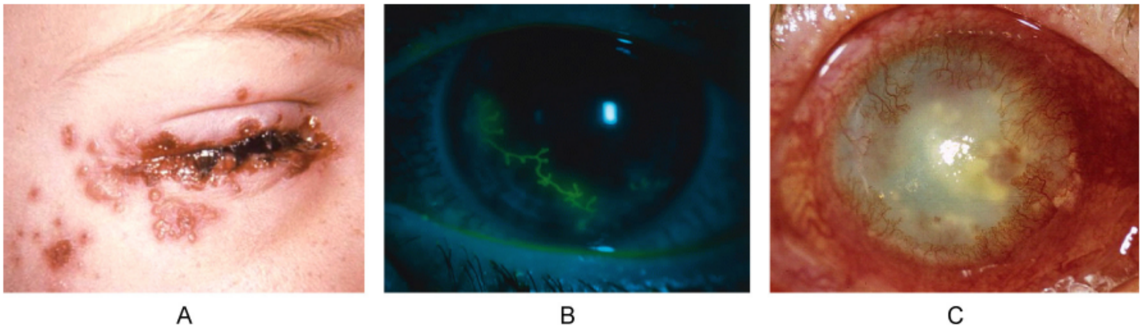

Figure 2. Clinical presentation of primary and recurrent ocular HSV infection. A: Typical HSV lesions on or about the eye lid margins (Source: CDC, Dr. K. L. Hermann). B: HSV infection can cause dendritic ulceration of the corneal epithelium, as shown here under fluorescein staining. These ulcers are characteristic of herpes epithelial keratitis (Tasman, 2013). C: Slit lamp photograph of patient presenting with stromal keratitis demonstrating corneal opacity and neovascularization (Rowe et al., 2013) (Reuse with permission).

producing an ocular HSV infection without prior skin or mucous membrane HSV infection.

## Clinical presentation

HSV can affect any part of the eye and cause pathology in all three layers of the cornea. This allows for a broad array of clinical manifestations with generalized symptoms such as tearing, redness, blurry vision and ocular discomfort. Primary HSV infection is usually asymptomatic and depends on the immunological status of the host. If present, it tends to manifest as conjunctivitis that can involve inflammation of the eyelids (blepharitis), ulcers and unilateral lid vesicles (Figure 2A) (Darougar S, et al., 1985; Rowe A M, et al., 2013). Corneal lesions from HSV may also arise, remaining on the epithelial layer, called infectious epithelial keratitis, or going deeper to involve the stroma, known as stromal keratitis.

Epithelial keratitis is the most frequent type of herpetic eye disease, accounting for 50%-80% of all ocular herpes infections (Wilhelmus K R, et al., 1981). HSV epithelial keratitis results from the virus replicating in and destroying the corneal epithelial cells. The earliest manifestation will start as small intraepithelial vesicles, which coalesce and grow into dendritic-shaped lesions with raised borders, central ulceration, and terminal end bulbs (Figure 2B) (Green L K, et al., 2006; Tasman W J E A, 2013). These dendritic ulcers are characteristic of epithelial keratitis and associated with pain, sensitivity to light, blurry vision, tearing and redness.

Keratitis with stromal involvement is seen more frequently in recurrent HSV infections, occurring in about 30% of those with epithelial keratitis (Labetoulle M, et al., 2005). Stromal keratitis can be further subcategorized into two types: necrotizing stromal keratitis and immune stromal keratitis. In necrotizing stromal keratitis, viral invasion causes dense infiltration, ulceration and necrosis in the stroma. There is however no defect in the epithelium. More commonly, stromal keratitis will present as the latter, immune stromal keratitis, which arises from recurrent HSV infection of the corneal stroma. Clinical signs include stromal opacity, edema and neovascularization, presumably triggered by the potent immune response to retained viral proteins after infection has cleared (Figure 2C). Corneal vascularization, in particular, is a prominent contributor to corneal damage in stromal keratitis. The ingrowth of vessels in the eye can interfere of the passage of light into the retina, deposit lipids and proteins into the corneal stroma, and serve as a passageway for inflammatory cells to enter the eye. The resultant damage to the structural integrity of the cornea leaves behind scar tissue after the inflammation clears (Gimenez F, et al., 2013). Repeated bouts of keratitis can lead to progressive irreversible stromal scarring and visual morbidity. For this reason, stromal keratitis is generally considered the most serious of the HSV ocular diseases.

Recurrent HSV keratitis can also present as endotheliitis (endothelial keratitis) or neurotrophic keratopathy (neurotrophic keratitis or NK). Endotheliitis occurs when the virus spreads to the corneal endothelium, the innermost layer of the cornea that is critical for maintaining corneal transparency. Inflammation of the cells here increases endothelial dysfunction and damages the aqueous pumps (Sundmacher R, 2009). Subsequent corneal edema without any signs of inflammation in the stroma is one indication of endotheliitis. Endotheliitis is also identified by the presence keratic precipitates, which are cellular deposits on the corneal epithelium caused by inflammation. The distribution of these precipitates and configuration of overlying stromal edema determines the classification between linear, sectorial, disciform and diffuse endotheliitis (Suzuki T, et al., 2008).

Neurotrophic keratopathy (NK), on the other hand, is a degenerative corneal disease characterized by a decrease or absence of corneal sensation. The disease has a multifactorial etiology, often appearing after a long history of dendritic ulcers and treatment with several antiviral agents that led to impairment of the trigeminal innervation, damage to the epithelial basement membrane and inflammation of the stroma. Clinically, patients with neurotrophic keratopathy present with symptoms that range from reduced tear film production and decreased visual acuity, to persistent corneal epithelial defects, ulcers, stromal opacification and corneal neovascularization. Mackie proposed three stages of neurotrophic keratopathy based on severity (Figure 3). Stage 1 can be identified by changes in the corneal epithelium accompanied by superficial punctate keratopathy and corneal edema. Stage 2 involves persistent epithelial defects with smooth, rolled edges and stage 3 is characterized by the presence of corneal ulcers with stromal melting or corneal perforation (Hamrah P, et al., 2012; Roy F H, et al., 2008; Sacchetti M, et al., 2014). HSV can also cause iridocyclitis, panuveitis, and acute retinal necrosis (ARN). However, these ocular manifestations are rare and do not usually occur in immunocompetent hosts.

One last note is that the clinical presentations of ocular HSV vary significantly in children and adults. In adults, epithelial keratitis is the most common ocular manifestation of HSV infection, whereas in children, stromal keratitis is far more likely to develop due to a heightened inflammatory response (Revere K, et al., 2013). In the largest study to date of pediatric patients with corneal HSV infections, Liu et al. found that corneal scars develop in up to 80% of pediatric patients with HSV keratitis. Furthermore, children are more at risk for refractive amblyopia caused by keratitis-induced astigmatism; the team reported an increase of more than two diopters in astigmatism in roughly 30% of pediatric HSV keratitis patients and a final vision of 20/40 or worse in 26% of their patients (Liu S, et al., 2012). All in all, children with HSV keratitis are likely to have poor visual outcomes and are at high risk for recurrence, corneal scarring and vision loss.

## Diagnosis of ocular HSV infection

Diagnosis of ocular HSV is primarily based on clinical findings. Diagnostic testing is seldom needed due to characteristic clinical features. However, laboratory testing is available to confirm clinical suspicion in cases that lack typical findings in addition to almost all suspected cases of neonatal herpes infection. In the lab, cells from the base of the lesion can be obtained and analyzed. The gold standard in laboratory diagnosis of HSV keratitis is viral isolation in culture. Other techniques can also be used for reliable, quicker diagnostic testing. A few for example are polymerase chain reaction (PCR) assays that detect viral DNA and enzyme- and fluorescence-based immunological assays that detect viral antigens. Despite having a lower sensitivity, the Giemsa stain can be utilized as well (Farhatullah S, et al., 2004). Herpesviruses distinctively fuse at neutral pH to form syncytia giving the appearance of giant multinucleated cells. As such, infected cells stained with Giemsa stain will be large in size and show the coalescence of infected cells and viral inclusion bodies.

## Management and treatment of ocular HSV

Primary ocular HSV will usually resolve by itself; nevertheless, proper treatment can shorten disease course, reduce severity and minimize damage, thereby lessening the long-term complications of HSV. Within in the last century, various physical, chemical and antiviral agents have been used to treat ocular HSV. These include curettage and cauterization in the 1890s, followed by corneal epithelial debridement, iodinisation, and carbolization in the first half of the 20th century (Wilhelmus K R, 2010). Many of these physiochemical interventions are still employed today. By means of physical scraping, chemical erosion and the application of cytotoxic reagents, these methods physically remove lesions and virus-infected

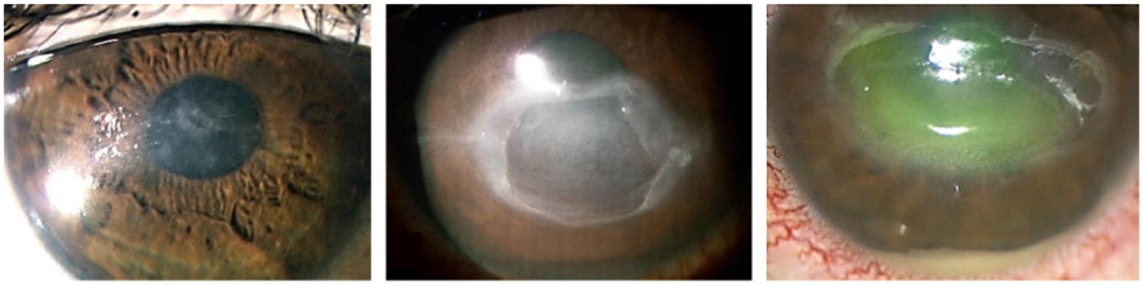

A B C

Figure 3. Three stages of neurotrophic keratopathy. A: Stage 1 NK with a cloudy and irregular corneal epithelium. B: Stage 2 NK with persistent epithelial defect with smooth and rolled edges. C: Stage 3 NK presenting with a deep corneal ulcer and stromal melting (Sacchetti and Lambiase, 2014) (Reuse with permission).

cells. The reduction in viral load aids in regeneration of the corneal epithelium to promote a faster recovery.

Researchers began to screen anti-infective compounds as potential treatments when it became clear that herpes viral infection was associated with epithelial keratitis. In 1962, idoxuridine, a pyrimidine analogue, became the first antiviral agent (Kaufman H E, 1962). Its success led to the development of other synthetic nucleoside analogues used in present day treatment of HSV. Now, antiviral agents are the mainstay of HSV therapy. Acyclovir (Zovirax) is one of the best-known antiviral drugs on today's market. The purine nucleoside analogue acts selectively against HSV without causing substantial toxic effects on uninfected cells. Antiviral nucleosides at large work by interfering with viral DNA synthesis during transcription of the viral genome to inhibit HSV replication. In the case of acyclovir, the drug is first phosphorylated into acyclovir monophosphate by the virus-encoded enzyme thymidine kinase, and then converted into its active form, acyclovir triphosphate, by human enzymes. Acyclovir triphosphate selectively inhibits viral DNA polymerase (Hung S O, et al., 1984). In doing so, the drug prevents viral replication, effectively reducing the severity of symptoms, frequency of viral shedding and spread of infection.

Current regimes to treat ocular herpes include a combination of physiochemical interventions and either topical or oral antiviral agents (Table 1). Antiviral treatment options include: ganciclovir ophthalmic 0.15% gel, trifluridine 1% drops, vidarabine 3% ointment, and oral acyclovir 400 mg, valacyclovir 500 mg, and famciclovir 250 mg. Because topical therapy can cause epithelial toxicity, they should be rapidly tapered after initial response and discontinued after complete healing. Oral therapy, on the other hand, has the advantage of no ocular surface toxicity and systemic antiviral activity (Hung S O, et al., 1984). As such, long-term use of an oral antiviral medication at a lower dose would be appropriate for patients experiencing recurrent ocular HSV.

A relatively new therapy-demonstrating efficacy in the treatment of HSV keratitis is the use of interferons. Interferons are antiviral proteins released by host cells immune system in the presence of viruses; two in particular, interferon-α and interferon-β, can activate an intracellular pathway to upregulate host genes that generate antiviral responses (Wilhelmus K R, 2010). Studies suggest that interferon therapy alone is as effective as treatment with a nucleoside antiviral agent; but a synergistic effect is observed when interferons are used in conjunction with an antiviral agent (Guess S, et al., 2007). In one study, treatment with interferon drops and acyclovir ointment led to an average resolution time of 3.9 days, as compared to an average resolution time of seven days using topical acyclovir alone (Colin J, et al., 1987). However, because human interferon is a biological compound, the high cost and need for refrigeration may limit its practicality.

Specific treatment plans will need to be evaluated depending on the presentation of the herpetic eye disease. Table 2 provides a general look at treatment strategies for varying ocular sequelae. Treatment for patients with epithelial keratitis aims to halt corneal infection in order to control symptoms and allow the normal ocular surface to become reestablished. The best evidence-based treatment of HSV epithelial keratitis seems to be trifluridine supplemented by ether topical or oral acyclovir (Wilhelmus K R, 2010). In cases of active herpetic epithelial keratitis, topical corticosteroids are contraindicated. This is because topical steroids inhibit normal immune defense mechanisms. The virus would then be able to replicate more quickly, thereby prolonging the course of infection and exacerbating disease severity.

Treatment for stromal keratitis employs topical corticosteroids accompanied by a prophylactic antiviral drug.

Table 1. Dosage of antiviral agents

| Oral antivirals | Dosage |
|---|---|
| Acyclovir | Treatment dose for adults: 400 mg 3-5x/day<br>Treatment dose for children: 12-15 mg/kg/day in several doses<br>Prophylactic dose: 400 mg 2x/day |
| Valacyclovir | Treatment dose: 500 mg 3x/day<br>Prophylactic dose: 500 mg once daily |
| Famciclovir | Treatment dose: 250 mg 3x/day<br>Prophylactic dose: 250 mg once daily or 125 mg 2x/day |
| **Topical antivirals** | **Treatment dosage** |
| Trifluridine 1% drop | One drop every 2 hours, reduced to 5x/day after 3-7 days |
| Ganciclovir 0.15% gel | 5x/day |
| Vidarabine 3% ointment | 5x/day |

Table 2. Treatment strategies for ocular HSV

| Ocular finding | Basic treatment approach |
|---|---|
| Epithelial keratitis | Topical or oral antiviral with possible debridement |
| Stromal keratitis without ulceration (immune stromal keratitis) | Topical steroid with prophylactic oral antiviral |
| Stromal keratitis with ulceration (necrotizing keratitis) | Oral antiviral in therapeutic doses with topical steroid |
| Endotheliitis (endothelial keratitis) | Oral antiviral in therapeutic doses with topical steroid |
| Neurotrophic keratopathy (neurotrophic keratitis) | Topical lubrication, soft contact lenses, tarsorrhaphy |

Even though stromal keratitis will spontaneously resolve itself within months, treatment can help prevent the otherwise profound stromal vascularization and scarring associated with the body's potent immune response to viral antigens. Corticosteroids, such as prednisolone, dampen the immune response by inhibiting CD4+ cell production of cytokines. In this manner, they combat the immunopathological component of stromal disease, while antiviral drugs thwart viral replication to shorten the duration of disease (Knickelbein J E, et al., 2009). The Herpetic Eye Disease Study determined that a tapering regimen of topical corticosteroid (1% prednisone for the first five weeks followed by 0.125% prednisone for the next five weeks) with a topical antiviral agent was able to significantly reduce the duration of stromal inflammation and progression of herpetic stromal disease (Barron B A, et al., 1994).

Similarly, endotheliitis is treated with a combined corticosteroid and antiviral therapy. For patients with neurotrophic keratopathy, no pharmacological treatments are available. Management of neurotrophic keratopathy relies on preservative-free artificial tears, autologous serum, and the use of corneal or scleral contact lenses to support corneal epithelial healing. Due to potentially detrimental effects on the ocular surface, all topical medications should be discontinued and any diseases relating to the ocular surface should be resolved. Surgical intervention may be necessary in cases of stromal thinning and perforation (Sacchetti M, et al., 2014).

## Vaccine development

Even with advances in antiviral therapy and the multitude of treatment options now available, recurrent infection and viral shedding are still not fully preventable. Thus, considerable effort has been expended towards the development of a vaccine against HSV. Vaccine development aims to produce a biologic agent that is either prophylactic or therapeutic. Prophylactic vaccines are designed to protect against primary HSV infection in those who are seronegative. They aim to contain the virus at the epithelial site of entry, thwart replication, and most importantly, block entry into the nerves. Protecting the nervous system from infection is especially important since the establishment of latency provides a source of intermittent reactivation and infection of the cornea. Therapeutic vaccines, on the other hand, are designed lessen the burden recurrent HSV infection in those who are seropositive and reduce the risk of transmission (Coleman J L, et al., 2013). These vaccines must be able to boost the existing immune effectors so that they stay above the threshold permitting virus reactivation (Pepose J S, et al., 2006). Because therapeutic vaccines enhance the immune response when reactivation occurs, they run the risk of inadvertently exacerbating corneal damage. With this in mind, a vaccine to prevent ocular HSV must be able to elicit an immune response that can control viral replication with minimal collateral damage to the cornea (Pepose J S, et al., 2006).

Since the 1920s, various HSV-1 and HSV-2 vaccines have been tested. Although some have shown promise in animal models, they fail to prove efficacy in human clinical trials. Specific hurdles in the development an effective vaccine include the virus's complex replication cycle, latent infection, immune evasion and large size (Azwa A, et al., 2009). To date, only one vaccine has exhibited partial efficacy against HSV. Developed by GlaxoSmithKline, the gD2-ASO4 vaccine (Simplirix) is a glycoprotein subunit vaccine. This vaccine was created from glycoprotein-D2 from HSV, an ideal antigen due to its essentiality in viral entry and ability to elicit a strong antibody response, and an ASO4 adjuvant consisting of aluminum hydroxide and 3-O-deacylated monophosphoryl lipid A to boost immune response.

Researchers tested the gD2-ASO4 vaccine in 2002 and observed benefits in women who were seronegative for both HSV-1 and HSV-2 antibodies at the time of vaccination. The vaccine was reported to be 73% protective of genital disease in these women, yet provided no protection for women who were seropositive for HSV-1 or HSV-2 and no protection for men (Stanberry L R, et al., 2002). Between 2002 and 2010, Belshe et al. further evaluated this vaccine's efficacy in the Herpevac Trial with a cohort of over 8,000 women screened to be seronegative for both viruses. They found that, surprisingly, the HSV vaccine only provided protection against genital disease and infection caused by HSV-1 (58% efficacy with two doses and 77% with three doses), but not against HSV-2 disease or infection (Belshe R B, et al.,

2012). While there are no obvious explanations for the gender difference in efficacy, it is postulated to be due to inherent anatomical differences in male and female genital mucosa. Vaginal tissues are surrounded by secretions containing antibodies and white blood cells which can act as an immunologic barrier, more effective at preventing infection than the mucosal immune system found in the epidermal tissues of men (Belshe R B, et al., 2012; Coleman J L, et al., 2013; Stanberry L R, et al., 2002). This study also suggests that a simple glycoprotein may not be adequately powered to induce a vigorous enough immune response to boost immunity in individuals previously infected with HSV.

Subunit vaccines are the most studied of HSV vaccines, but other approaches have also been explored. One example is the development of a live attenuated vaccine. In theory, live attenuated vaccines hold clear advantages over other methods. They can present the full range of viral antigens to the host immune system and bring about a broader, longer-lived immune response by stimulating both the humoral and cell-mediated immune responses as well as innate immunity (Nguyen L H, et al., 1992). Live attenuated vaccines must be able to sufficiently replicate in the host to evoke a robust immune response, but not cause the pain and ulcerative lesions characteristic of natural infection. Ideally, the vaccine candidate should also be neuroattenuated and retain its ability to establish latent infection. This is valuable as subclinical reactivation will periodically re-stimulate the immune system to generate a more effective immune response (Arvin A M, 2007). The disadvantage to live attenuated vaccines however is their potential to revert back to the wild phenotype. Other vaccine strategies currently being investigated are replication defective vaccines, peptide vaccines, DNA vaccines and live vector vaccines.

Right now, no successful vaccine against HSV-1 or HSV-2 has been created. The development of a subunit vaccine that affirms protection against HSV-1 genital disease is a step forward, but additional research is still needed. A better understanding of factors and their mechanisms affecting vaccine efficacy, such as gender differences and preexisting HSV-1 serological status, will help scientists overcome the challenges posed by HSV to traditional vaccine strategies and hopefully lead to the advent of an effective HSV vaccination.

## VARICELLA ZOSTER VIRUS

### Background

The varicella zoster virus (VZV) is the causative agent of two clinically distinct forms of disease. Primary infection presents as varicella, commonly known as chickenpox, and leads to a lifelong infection of the sensory ganglia neurons. Chickenpox is a highly contagious but generally benign childhood disease that presents with itchy blister-like rashes all over the body. Reactivation of latent VZV later in life results in herpes zoster, or shingles. Shingles usually manifests as painful unilateral vesicular eruptions, most often affecting the dermatomal distributions of the thoracic nerve (56%) and trigeminal nerve (15%) (Gross G, et al., 2003). The ocular manifestations of VZV infection can be divided into the ophthalmic sequelae of varicella and the ophthalmic sequelae of herpes zoster. Although varicella infection can create problems in the eye, severe pain and ocular complications typically stem from herpes zoster infection instead.

Unlike HSV, immunization against VZV does exist and is commercially available. In 1995, Varivax was the first VZV vaccine implemented in the United States. The live attenuated vaccine was developed using the Japanese varicella strain, Oka, and has decreased the number of hospitalizations and deaths from VZV by more than 90% (Prevention C f D C a, 2012). Its counterpart, the shingles vaccine (Zostavax), was approved in 2006 for individuals over the age of 50. This vaccine also uses the VZV Oka strain, but is about 15 times stronger and has a much larger dosage of virus (Gershon A A, et al., 2010). Despite demonstrating efficacy in lessening the morbidity associated with chickenpox and shingles, these vaccines have also permanently changed the epidemiology of VZV-elicited diseases the United States.

### Varicella

Varicella, or chickenpox, is a highly infectious disease that developed in virtually all children prior to the introduction of the varicella immunization. In fact, in the pre-vaccination era, there were an estimated 60 million new cases of chickenpox worldwide each year, and roughly 95% of the population had serological evidence of prior a VZV infection (Papaloukas O, et al., 2014).

VZV can be found in respiratory secretions and fluid from cutaneous lesions. The virus typically spreads via respiratory droplets, although transplacental transfer of VZV can also take place if the mother acquires varicella during early pregnancy (Arvin A M, 1996). In most cases, the virus enters the host through the respiratory mucosa. During an incubation period of 10-21 days, VZV initially replicates in the upper respiratory tract followed by primary subclinical viremia. The virus is transported back to the respiratory system towards the end of the incubation period, allowing for the transmission of VZV to susceptible individuals before onset of the characteristic chickenpox rash (Papaloukas O, et al., 2014). Clinical symptoms begin to appear after additional viral replication and a second viremic phase. In immunocompetent patients, humoral antibodies will quickly eliminate the virus from the bloodstream while cellular lymphocyte-mediated immune response handles intracellular virus. Despite this, some VZV still spreads to sensory ganglia throughout the body and establishes latency. Subsequent reactivation of the virus then produces the other distinct viral syndrome, herpes zoster.

The main clinical manifestations of varicella are fever, malaise and mucocutaneous exanthem with itchy vesicular eruptions of the skin. These vesicles take about one week after their initial appearance to rupture and become encrusted, at which time the patient is no longer contagious. Ocular manifestations accompanying varicella are most commonly mild conjunctivitis and episcleritis (Liesegang T J, 2008). Epibulbar phlyctenule-like lesions can also form on the cornea, referred to as pocks. These are round, focal accumulations of lymphocytes and inflammatory cells that often appear during active systemic varicella and suspected to harbor live virus. They can also emerge months later as an immunologic reaction to retained viral antigens. In either case, the pocks are usually mild and persist for one to two weeks before resolving on their own. Less frequently, varicella may present as punctate or dendritic epithelial keratitis, nummular keratitis, and stromal keratitis. Keratitis from VZV closely resembles herpes simplex keratitis, but with a few distinguishable features (Figure 4). Firstly, VZV dendrites do not leave ulcerated lesions as seen with herpes simplex. These pseudodendrites tend to be smaller, polymorphous and elevated, with less distinct branching patterns and lack terminal bulbs (Tyring S K, 2006). In addition, VZV keratitis usually does not recur and is less responsive to topical antiviral therapy.

Generally speaking, varicella is a self-limiting disease that requires minimal treatment. Oral acyclovir can be used to treat varicella in otherwise healthy children, but its administration is controversial since the drug has not demonstrated a significant reduction in varicella-related complications or transmission (Kim S R, et al., 2014). In neonates and immunocompromised individuals, varicella infection can bring about severe complications such as pneumonitis, encephalitis, optic nerve atrophy, and cataract. Treatment with intravenous acyclovir in these patients will substantially decrease their disease morbidity, progression, and mortality (Prober C G, et al., 1982).

## Herpes zoster

With over one million new cases each year in the United States alone, herpes zoster (shingles) is a major public health concern in both the US and worldwide. The disease ensues when latent VZV reactivates from the sensory ganglia and spreads to the corresponding dermatome. Age is the most common predisposing factor to developing zoster, as changes in T cells and a decrease in neutralizing VZV antibodies allow for the latent virus to reactivate (Kahloun R, et al., 2014). Aside from the immunosenescence of aging, immune suppression from possible malignancy or immunosuppressive therapy is also linked to a higher risk of herpes zoster. In fact, herpes zoster is four to five times more likely in patients who are immunocompromised than in those who are not.

While herpes zoster is rarely fatal, the accompanying morbidity and burden are substantial. Nearly 50% of older adults with herpes zoster will experience complications (Oxman M N, et al., 2005). The most frequent and debilitating of these is postherpetic neuralgia (PCN), a chronic neuropathic pain syndrome that can persist for months, years or even life (Schmader K, et al., 2008). Representing an estimated 10%-20% of all zoster cases, herpes ophthalmicus (HZO) is the second most common complication. Although zoster can involve any division of the trigeminal nerve, it is 20 times more likely to affect the ophthalmic branch (Tyring S K, 2006). HZO is the term used to describe a zoster infection with involvement of the ophthalmic branch of the trigeminal

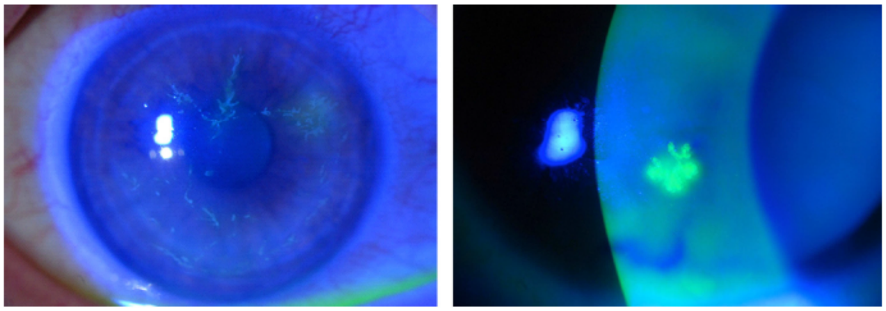

A B

Figure 4. VZV keratitis and HSV keratitis with fluorescein stain. A: VZV pseudodendrites are smaller and do not have terminal end bulbs. They are formed by heaped up epithelial cells that only stain mildly with fluorescein stain. Courtesy of Dr.Rapuano.(Source: http://dnn.testshell.net/Images.aspx). B: HSV dendrites are true dendrites. They cause ulceration with terminal end-bulb formations. The ulcer base stains vividly with fluorescein dye. Courtesy of Dr.Rapuano.(Source: http://dnn.testshell.net/images/corneal_04.jpg).

nerve, regardless of any intraocular association. In a cohort study examining trends in herpes zoster over a 28-year period, Yawn et al. discovered that the rate of eye complications in individuals with herpes zoster increased 23% between 1970 and 2007. They also found that these eye complications result in considerable health care usage, with an average of more than eight visits over a six-month or longer period (Yawn B P, et al., 2013).

Ocular manifestations can occur concurrently or following infection due to a delayed cell-mediated hypersensitivity reaction to viral DNA antigens, which can be found in the cornea up to 10 years after the initial episode of zoster (Wenkel H, et al., 1998). Most cases of HZO begin with a prodrome of severe pain and hyperesthesia over the affected dermatome, mild fever and malaise. After a few days, cutaneous lesions usually emerge. The initial HZO eruption may give the appearance of small raised bumps on the skin, but will become vesiculopapular and ulcerative before the lesions rupture and crust over, requiring several weeks to heal completely (Shaikh S, et al., 2002). As the inflammation resolves, permanent scarring with loss of normal pigmentation may occur. Although a majority HZO cases show this classic rash, a small number of patients will present with only HZO-like ophthalmic findings without any dermatomal lesions. This is called zoster sine herpete (Gilden D H, et al., 1992).

Inflammation from HZO can affect all ocular and adnexal tissues. As such, a wide and complex array of sequelae is associated with HZO. The clinical spectrum of HZO findings is presented below in Table 3. HZO can involve the eyelids, conjunctiva, sclera, episclera, cornea and iris, with the most frequent ophthalmic manifestations being keratitis (76.2%), uveitis (46.6%) and conjunctivitis (35.4%) (Yawn B P, et al., 2013). On the eyelids, HZO will produce vesicular lesions in the majority of patients that tend to resolve with minimal scarring. Conjunctivitis often occurs and will resolve in one week, although secondary infections can develop. Corneal complications are seen in about 65% of HZO cases. Unlike involvement of the eyelids or conjunctiva, corneal involvement can cause significant visual morbidity from pain, light sensitivity and vision decrement due to corneal neovascularization, thinning, and perforation. Another serious complication of HZO is acute retinal necrosis (ARN). In addition, a more extreme version, progressive outer retinal necrosis, is observed in immunocompromised zoster patients, especially those who also have HIV (Wong R W, et al., 2013). Both conditions can lead to retinal detachment and blindness, but the prognosis for patients with progressive outer retinal necrosis is especially poor.

Most HZO cases are mild and usually result in short-term inflammation that resolves without topical antiviral treatment. Nonetheless, treatment with antivirals within the first 72 hours after rash onset is strongly recommended. Not only can early treatment shorten the duration of disease, it can also ease acute pain, diminish severity and reduce the percentage of eye disorders in HZO patients from 50% to 20%-30% (Cobo L M, et al., 1986). Widely prescribed antivirals are acyclovir, valacyclovir, and famciclovir. Although all three drugs have similar efficacy in treating HZO, the newer drugs, valacyclovir and famciclovir, have the advantage of a simpler dosing regimen, higher bioavailability and the ability to decrease the incidence and severity of PHN (Tyring S K, 2007). Corticosteroids can also be applied in the first few weeks with antiviral therapy to offer additional pain relief and improve cutaneous healing. Long-term corticosteroid use should be avoided due to adverse effects and steroids in general should not be used for the treatment of epithelial

Table 3. Ocular Manifestations in Herpes Zoster Ophthalmicus

| Structure involved | Acute phase | Late phase |
|---|---|---|
| Eyelid/conjunctiva | Blepharitis | Ptosis |
| | Conjunctivitis | Eyelid retraction |
| | Vesicular rash | Secondary Staphylococcus aureus infection |
| Episclera/sclera | Episcleritis | Self-limiting |
| | Scleritis | Focal sectoral atrophy |
| Cornea | Epithelial (punctate or dendritic) keratitis | Neurotrophic keratopathy |
| | Stromal keratitis | Corneal neovascularization |
| Anterior chamber | Uveitis | Focal iris atrophy |
| | | Secondary glaucoma |
| | Ocular hypertension | Usually self-limiting |
| Retina | Retinal vasculitis | Acute retinal necrosis |
| | Retinitis | Progressive outer retinal necrosis |
| Cranial nerves | Optic neuritis | Optic atrophy |
| | Oculomotor muscle palsies | Self-limiting |

* Adapted from Opstelten W, et al.(2005), Rocha G, et al.(2010), and Shaikh S, et al.(2002).

keratitis as mentioned previously. Late HZO manifestations do not respond as well to antiviral drugs. Rather, they require topical steroids to reduce inflammation with patients usually remaining steroid-depend for long periods of time. Table 4 provides a more comprehensive look at treatment strategies for specific HZO complications.

In theory, the development of cell-mediated immunity to VZV from infection protects an individual from future episodes of varicella and zoster. However, VZV can still reactivate when this immunity is no longer as strong, such as in circumstances of immune suppression or from the natural decline in immune status with age. Preventing herpes zoster thus requires routine immune "boosting" in order to keep the cell-mediated immunity primed and effective. This generally occurs from two sources: periodic, asymptomatic, release of VZV from the ganglia and exposure to varicella-infected individuals. With the gradual disappearance of wild-type VZV after the introduction of the varicella vaccine though, fewer adults are getting re-exposed to the virus, causing their immunity to decline. Because of this, an increase in the incidence of herpes zoster was expected in the years following varicella vaccination, but this has not yet been observed (Liesegang T J, 2008; Papaloukas O, et al., 2014).

The herpes zoster vaccine, Zostavax was intended to substitute for the boosting effect no longer provided by vaccinated children. The vaccine's ability to adequately heighten a person's immunity against VZV was affirmed by Weinberg et al. Moreover, they found greater VZV cell-mediated immune responses at onset of zoster to be correlated with reduced severity of disease and lower occurrence of PCN, demonstrating the protective effect of cell-mediated immunity against zoster morbidity (Weinberg A, et al., 2009). Unfortunately, this increased immunity can also target persistent viral DNA in ocular tissues of individuals with prior HZO and provoke the recurrence of ocular manifestations (Hwang C W, Jr., et al., 2013). As such, care should be taken in administering the Zostavax vaccine in patients with a history of HZO to avoid the possibility of a vaccine-induced reactivation. All in all, the zoster vaccine is generally safe and well tolerated (Simberkoff M S, et al., 2010). The pivotal Shingles Prevention Study indicated that Zostavax not only lowered the overall incidence of herpes zoster by 53.1%, but also substantially decreased the pain and discomfort in those who did develop zoster, thereby reducing the burden of illness associated with this debilitating disease (Keating G M, 2013; Oxman M N, et al., 2005).

## CYTOMEGALOVIRUS

### Background

Cytomegalovirus (CMV) is another herpesvirus that can affect the eyes. The species of CMV that infect humans is known as human CMV or human herpesvirus-5 (HHV-5). Although the virus is found in all geographic locations, its prevalence depends on socio-economic status, infecting 60%-70% of individuals in industrialized countries and virtually 100% of individuals in emerging countries (Fulop T, et al., 2013). CMV infection can

Table 4. Treatment for Herpes Zoster Ophthalmicus

| Ocular finding | Treatment |
|---|---|
| Skin and lid vesicular lesions<br>Pain and hyperesthesia of ophthalmic branch dermatome | Oral acyclovir (800 mg 5x/day) for 7-10 days, valacyclovir (1000 mg 3x/day) for 7 days, or famciclovir (500 mg 3x/day) for 7 days |
| Epithelial keratitis | Gentle debridement or no treatment |
| Stromal keratitis | Topical corticosteroid and cycloplegic agent |
| Neurotrophic keratitis | Topical lubrication<br>Topical antibiotics for secondary infections<br>Protective contact lenses to prevent corneal perforation<br>Tarsorrhaphy |
| Uveitis | Topical steroids<br>Oral steroids<br>Oral acyclovir |
| Episcleritis/scleritis | Topical nonsteroidal anti-inflammatory agents and/or steroids |
| Retinitis<br>Acute retinal necrosis<br>Progressive outer retinal necrosis<br>Cranial nerve involvement | Intravenous acyclovir (1500 mg per m2/day divided into 3 doses) for 7-10 days followed by oral acyclovir (800 mg, 5x/day) for 14 weeks<br>Systemic steroids<br>Laser/surgical intervention if needed |

* Adapted from Shaikh S, et al.(2002), Tyring S K, (2006).

yield serious consequences in those with weakened immune systems. It is the most significant viral cause of birth defects in developed countries and considered an AIDS-defining infection in HIV patients; indicative of T-cell counts dropping to low levels (Damato E G, et al., 2002).

CMV can be found in body fluids such as the blood, saliva, urine, tears, semen and breast milk of an infected person. The virus is transmitted via close contact and through receiving transplanted organs or transfusions of blood. Primary CMV infection is clinically silent in most individuals with normal immune function. Some may develop a mononucleosis syndrome, presenting with malaise, headache and high fever that can persist for weeks, but is usually mild and self-limiting. The virus will then enter a latent state and is continually suppressed by cell-mediated immunity in an otherwise healthy individual. Only when an abrogation in immune responses occurs will the virus reactivate to cause problems. Primary CMV infection in immunocompromised patients on the other hand, manifests as a wide range of conditions. These patients usually have immature or impaired immune systems, for example HIV-infected individuals, organ transplant recipients, and newborn infants, with the severity of disease typically proportional to the degree of immunosuppression (Varani S, et al., 2011). CMV infection in this population can result in CMV pneumonia, hepatitis, encephalitis, retinitis, seizures, coma, visual impairment and other problems.

## Ocular manifestations

Retinitis is the most common illness caused by CMV, although the virus is responsible for other ocular manifestations as well, including anterior uveitis, corneal endotheliitis, and ARN. CMV retinitis is a vision-threatening opportunistic infection that arises in those who are immunosuppressed and in infants with congenital CMV. Before 1982, cases of CMV retinitis were extremely rare (Hennis H L, et al., 1989). With the onset of the AIDS epidemic, its incidence has significantly risen and is now the most common cause of vision loss in AIDS patients. In addition, CMV retinitis is occurring more frequently in the general population due to the increased use of new immunosuppressive drugs and emerging as a regular cause of infectious retinitis around the world (Bloom J N, et al., 1988; Hennis H L, et al., 1989).

CMV invades the retina through blood vessels and produces retinal lesions and hemorrhages. Without treatment or improvement in immune function, the virus will spread to adjacent areas of healthy retina, causing damage to the retina as well as the optic nerve, and resulting in necrosis, retinal detachment, and complete vision loss in just two to six months (Tyring S K, 2006). When the vitreous is involved, symptoms may initially present as blurred vision with the onset of floaters. This often starts in one eye and progresses to the other. If the macula becomes involved, symptoms will include a rapid loss of visual acuity and visual perception (Hennis H L, et al., 1989).

Under ophthalmoscopic examination, CMV retinitis has a characteristic appearance. Areas of active retinitis, caused by necrosis and edema, will look granular and cloudy-white in color. Retinal hemorrhages can also be seen, which occur as the virus attacks the endothelial cells of blood vessels. Fundus photographs of CMV retinitis depict one of two presentations (Figure 5). Some cases will show diffuse areas of white infiltrates that are usually close in proximity to a major retinal vessel or the optic nerve (Figure 5A) (Palestine A G, 1988). This juxtaposition of white, granular zones of necrosis with regions of red retinal hemorrhage led to the classic "pizza pie" or "cottage cheese with ketchup" description. In other cases, patients may present with one or two focal granular infiltrates with variable amounts of associated hemorrhage (Figure 5B). As these lesions slowly enlarge, they take on a brushfire appearance and leave behind

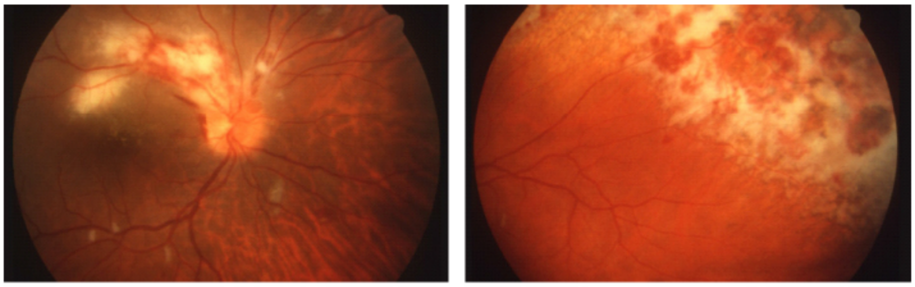

A B

Figure 5. The fundus photograph of CMV retinitis is distinct. A: CMV retinitis with the classic mixture of retinal infection (white zones) and hemorrhage (red spots). B: Extensive retinitis in a brushfire pattern. The upper right region shows retinal necrosis and atrophy that changed the retinal pigment epithelium, producing a dark pigmented appearance (Anderson et al., 2013) (Reuse with permission).

necrotic retina and atrophic retinal pigment epithelium (Tasman W J E A, 2013).

Despite the gravity of this disease, CMV retinitis usually progresses slowly, meaning a large window of opportunity exists to provide treatment and hopefully thwart vision loss (Bowen E F, et al., 1996). Treatment of CMV retinitis uses antivirals in conjunction with highly active antiretroviral therapy (HAART). Valganciclovir, ganciclovir, foscarnet, and cidofovir are antiviral medications that can be administered orally, intravenously or directly injected into the eye (Stewart M W, 2010). Additionally, ganciclovir implants are another available option. These devices are implanted into the eye and release the drug at a rate of 1μg/hr for up to nine months. Studies have indicated that the sustained-release ganciclovir implant is actually more effective at treating CMV retinitis than intravenous ganciclovir (Musch D C, et al., 1997).

HAART therapy is a combination of antiretroviral medications whose widespread use has led to a significant reduction to the incidence of opportunistic infections, including that of primary and recurring CMV retinitis (Varani S, et al., 2000). HAART-induced immune recovery not only lessens the incidence of CMV retinitis by an estimated 80%, but also provides retinitis patients with a lower risk of progression, retinal detachment, visual field loss and mortality. One thing to note though is that the benefits of HAART therapy are attenuated in CMV retinitis patients experiencing immune reconstitution. As the immune system grows stronger from antiretroviral therapy, covert infections may become unmasked and overt infections can be exacerbated (Tappuni A R, 2011). In CMV retinitis patients whose immune status improves with HAART therapy, immune recovery uveitis will often surface in its place (Nguyen Q D, et al., 2000) (Whitcup S M, 2000).

Even with treatment, CMV retinitis can still worsen and lead to blindness. Furthermore, all anti-CMV drugs currently licensed have limitations, in particular, the development of drug resistance and toxicity (Schreiber A, et al., 2009). No CMV vaccines exist to date, although a few vaccine candidates are under investigation. One noteworthy candidate in Phase II trial is a recombinant glycoprotein-B subunit vaccine with a reported overall efficacy of 50% in seronegative women of childbearing age (Sung H, et al., 2010).

## OTHER HERPESVIRUSES

### Epstein-Barr virus

The Epstein-Barr virus (EBV) is the most common causative agent of infectious mononucleosis and associated with certain forms of cancer, such as Hodgkin's lymphoma, Burkitt's lymphoma, and nasopharyngeal carcinoma (Matoba A Y, 1990). The virus is usually transmitted by contact with saliva and infects B-lymphocytes during primary infection. On rare occasion, EBV is connected to a variety of ocular infections encompassing all segments of the eye. These manifestations are attributed to the virus largely on the basis of observational associations and seroepidemiologic data, as its genome has been detected in all ocular tissues except the optic nerve (Slobod K S, et al., 2000). The most common are periorbital edema and follicular conjunctivitis (Newman H, et al., 2013). Presentations of corneal epithelial and stromal infections have also been linked to EBV, as well as reports of EBV-induced episcleritis, keratitis, retinitis and uveitis. In addition, EBV has also been implicated in ocular tumors.

Management of EBV infections in the eye is not well established since the mechanisms of disease production are still unclear. As a whole, treatment is primarily supportive. Conjunctivitis tends to resolve spontaneously and in other ocular scenarios, analgesia, lubricants and topical corticosteroids can be applied. Antiviral and interferon-α therapy have also been used to treat cases of EBV-associated ocular diseases, but met with limited success.

### Herpesvirus-6 and herpesvirus-8

Far less frequently, herpesvirus-6 (HHV-6) and herpesvirus-8 (HHV-8) may elicit ocular complications. HHV-6 infects almost all children before the age of 2 and is associated with febrile illnesses, immunodeficiency disorders and neurological diseases (Caserta M T, et al., 2001). Very rarely, HHV-6 can cause optic neuritis and possibly dry eye and papillary conjunctivitis (Farooq A V, et al., 2010; Mechai F, et al., 2007; Ogata N, et al., 2011). HHV-8, also referred to as Kaposi sarcoma-associated herpesvirus (KSHV), can result in ocular Kaposi's sarcoma and perhaps uveitis (Daibata M, et al., 2000; Matsuo T, et al., 2002). Kaposi's sarcoma affects ocular structures in about 20%-30% of AIDS patients. Although other ocular adnexa can be involved, Kaposi's sarcoma of the eye is typically found on the eyelid or conjunctiva (Tyring S K, 2006). Fortunately, ocular Kaposi's sarcoma is rarely vision threatening and can be treated with cryotherapy, surgical excision, radiation, and chemotherapy.

## CONCLUSION

Herpesviruses are a major contributor of infectious disease in humans and an important cause of ocular morbidity. Most cases of herpetic eye disease are associated with the herpes simplex virus, varicella zoster virus, and cytomegalovirus, although a few other herpesviruses have also been implicated in ocular disease. While these viruses asymptomatically infect a large proportion of the human population, their ability to establish latency with persistent lifelong infections is particularly troublesome. Recurrent ocular herpes infections account for the mass majority of visual morbidity. They can wreak devastating effects and lead to irreversible vision loss accompanied by a decreased quality of life, increased healthcare usage, and significant cost burden.

Antiviral medications and new therapies have expanded treatment options for these patients. Such interventions can reduce the recurrence of disease, mitigate symptoms and lessen the long-term complications of herpetic eye disease. However, no treatment currently exists to eradicate herpesviruses from the body after primary infection. Furthermore, the array of therapeutic strategies only underscores the lack of any one successful treatment. The advent of effective vaccines will be critical to preventing herpesvirus diseases altogether and cutting the incidence of ocular complications. To date, only anti-VZV vaccines have been developed. Continuing research on the vaccine front has thus far provided a greater understanding of the complexities of herpesviruses, but more progress is needed before other herpesvirus vaccines can be approved for general use.

## COMPLIANCE WITH ETHICS GUIDELINES

All the authors declare that they have no competing interests. This article does not contain any studies with human or animal subjects performed by any of the authors.

## OPEN ACCESS

This article is distributed under the terms of the Creative Commons Attribution License which permits any use, distribution, and reproduction in any medium, provided the original author(s) and thesource are credited.
